# Development of the Myasthenia Gravis (MG) Symptoms PRO: a case study of a patient-centred outcome measure in rare disease

**DOI:** 10.1186/s13023-021-02064-0

**Published:** 2021-10-30

**Authors:** Sophie Cleanthous, Ann-Christin Mork, Antoine Regnault, Stefan Cano, Henry J. Kaminski, Thomas Morel

**Affiliations:** 1 Modus Outcomes, Letchworth Garden City, UK; 2 UCB S.A., Allée de la Recherche, 60, 1070 Brussels, Belgium; 3 Modus Outcomes, Lyon, France; 4 George Washington University, Washington, DC USA; 5 KU Leuven, Leuven, Belgium

**Keywords:** Myasthenia gravis, Rare disease, Outcome, Patient-centred, Mixed methods psychometrics

## Abstract

**Background:**

Myasthenia gravis (MG) is a chronic autoimmune neuromuscular disease, characterised by fluctuating muscle weakness which makes it challenging to assess symptom severity. Mixed methods psychometrics (MMP), which combines evidence from qualitative research and modern psychometrics, is a versatile approach to the development of patient-centred outcome measures (PCOM) in the context of rare disease. Our objective was to develop the MG Symptom patient-reported outcome (PRO) to assess key aspects of MG severity from the patient perspective.

**Methods:**

We used MMP to develop a novel PRO instrument in a multi-step process. An initial conceptual model for MG patient experience was developed and expanded based on preliminary literature review and two waves of concept elicitation interviews with people with MG (Step 1). Based on this, the novel PRO instrument, the MG Symptoms PRO, was drafted. The draft instrument was refined by combining qualitative and quantitative data collected in a Phase 2 clinical study (Step 2).

**Results:**

Findings from the literature review and concept elicitation interviews (n = 96) indicated that patient experience in MG includes proximal muscle weakness symptoms related to several body parts, along with muscle weakness fatigability and general fatigue. Then, a set of 42 items across five scales (ocular-, bulbar-, and respiratory muscle weakness, physical fatigue, and muscle weakness fatigability) was developed. Qualitative evidence endorsed its relevance, clarity, and ease of completion; quantitative analysis with Rasch measurement theory methods demonstrated strong measurement properties, including good targeting and high reliability. Classical test theory analyses showed adequate reliability of the instrument and mild to moderate correlations with other widely used MG-specific outcome measures.

**Conclusions:**

The MG Symptoms PRO has potential to be used both to measure treatment benefit in clinical trials and monitor symptom severity in clinical practice. Its component scales were purposefully designed to stand alone, enhancing interpretability of scores given the heterogeneity of MG, and enabling modular use. Compared with existing MG PROs, it contains more detailed assessments of muscle weakness and muscle weakness fatigability symptoms, which are of key importance to people with MG. The MMP approach used may serve as a case study for developing PCOMs across rare disease indications.

**Supplementary Information:**

The online version contains supplementary material available at 10.1186/s13023-021-02064-0.

## Introduction

Patient-centred outcome measures (PCOMs) are essential for demonstrating that treatment effects translate into a clinical benefit that is meaningful to patients. PCOMs are powerful tools because they focus on issues that matter most to patients, ensuring that their experiences are accurately reflected in clinical research and practice. Unfortunately, for many rare disease indications PCOMs are either not available or not widely used [1]. Development of patient-reported outcome (PRO) instruments in the context of rare disease is challenging, because low prevalence limits the number of patients available to participate in PRO development efforts, and because heterogeneity of symptom presentation, disease severity and progression often further complicates the process [1–3].

In the recent IRDiRC Orphan Drug Development Guidebook, PCOMs were identified as a ‘building block’ for orphan drug developers, and their use is encouraged as efficacy endpoints in clinical trials, outcome measures in registries, or tools to monitor care delivery. The Guidebook outlines several requirements for developing a PCOM, including the generation of extensive patient evidence (preferably through mixed methods research) and psychometric data, multi-stakeholder collaboration and, for developing de novo PCOMs in particular, regular scientific advice from regulatory bodies [4].

Myasthenia gravis (MG) is a rare, clinically heterogeneous autoimmune neuromuscular disease [5] with an estimated annual incidence of 1 in 500,000 people in the US and an estimated prevalence of between 1 in 2500 and 1 in 200,000 people [6]. In Europe, it is estimated that 2 in 10,000 people are affected by MG [7]. MG is caused by the production of pathogenic IgG autoantibodies against neuromuscular junction components (AChR, MuSK and LRP4) and manifestation can be generalised (gMG), affecting bulbar, limb and respiratory muscles [8], or limited to ocular (oMG), where weakness is confined to extraocular muscles [9]. Most people present with oMG, but 80–85% of cases progress to gMG [10, 11].

gMG is characterised by fluctuating and variable muscle weakness, muscle fatigability (i.e., triggering or worsening of an impairment with usual or normal activities, or onset/worsening of an impairment over the course of the day) and generalised fatigue (i.e., becoming increasingly tired). Symptoms contribute differently to the degree of clinical disability [12] but collectively impact many aspects of the quality of life of people living with MG. These range from physical exertions (e.g., walking and doing housework), social activities, sleep, psychological health and professional development [13–15]. In cases where muscle weakness extends to the respiratory muscles, the condition may become life-threatening [8].

Due to this fluctuating and unpredictable disease course and the subjective nature of symptoms such as fatigue, PRO instruments have the potential to provide greater insight into the experience of people living with MG than traditional clinical endpoints, and regulators are encouraging their use as primary efficacy trial endpoints [12]. However, the heterogeneity of the disease can lead to a lack of correlation between some clinical measures at onset and remission or worsening episodes (e.g., the Myasthenia Gravis Foundation of America [MGFA] clinical classification) [16]. Robust PCOM and PRO instruments genuinely grounded in the patient experience would complement the currently widely-used clinician-reported measures that aim to quantify the severity of MG based on impairments to body functions, such as Quantitative Myasthenia Gravis Score (QMG) and Myasthenia Gravis Composite (MGC) [17, 18].

Multiple PRO instruments have been developed to capture the impact of MG on an individual’s life, including the Myasthenia Gravis Activities of Daily Living (MG-ADL) [19], MG Disability Assessment (MG-DIS) [14], the MG Fatigue Scale (MGFS) [20], and the MG Quality of Life 15 (MG-QoL-15) [21]. However, these PRO instruments may not comprehensively assess the range of symptoms and functional impact proximal to the MG experience [12] and fatigability, in particular, is often overlooked [22]. Additionally, these PRO instruments largely fail to meet the latest regulatory and expert recommendations in relation to having patients involved in the development process [4, 23, 24].

Mixed methods psychometrics (MMP) is an approach that can be used in the development of PCOM instruments. MMP combines evidence from both qualitative and quantitative sources in an iterative process, on the premise both evidence sources are essential, but neither sufficient independently. As such, MMP is ideal for patient-centred research, as it encompasses both patient experience and feedback throughout the instrument development process to ensure that the PRO item content is important and relevant to patients [25]. The versatility of MMP is valuable in challenging contexts such as rare disease, where trial cohorts are small, as it maximises the amount of evidence that can be used in the PCOM development process [1].

The objective of this paper is to describe the development of a new PRO instrument, the MG Symptoms PRO, which was developed and validated using state-of-the-art MMP psychometric analyses, including interviews with over 90 patients and examination of measurement performance in people living with MG. Development of the MG Symptoms PRO can be used as a test-case for developing PCOMs in rare disease.

## Methods

### Study design and data collection

**Fig. 1:**
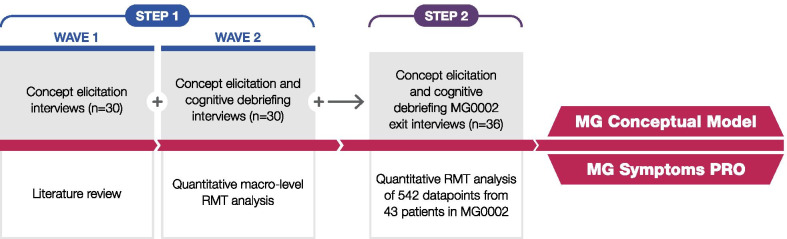
Study design for development of the MG Symptoms PRO, a new PCOM in rare disease. *MG* myasthenia gravis, *PCOM* patient-centred outcome measure, *PRO* patient-reported outcome, *RMT* Rasch measurement theory

We performed a two-step MMP study (Fig. 1). Step 1 involved a literature review and two waves of interviews with people with MG, leading to the development of a preliminary conceptual model of the patient experience in MG and draft items for the new PRO instrument. In Step 2, the analysis of qualitative and quantitative data from the phase 2 clinical trial MG0002 [26], in which the draft items were tested, led to the refinement of the conceptual model and draft item set. The study provided qualitative evidence from participant exit interviews and qualitative evidence from the analysis of the draft items in line with RMT. Additionally, supportive evidence on measurement properties was generated in the MG0002 study cohort sample using CTT. Feedback from clinical experts with more than 20 years of experience in treating MG was sought to inform interpretation of results and decision making.

#### Step 1: Literature review and two waves of interviews

The literature review appraised qualitative studies related to the patient perspective of disease experience in MG and disease-specific PRO instruments. Analysis of the reviewed qualitative studies led to the development of a preliminary conceptual model. The content of available PRO instruments was further mapped onto the conceptual model to examine the coverage of these instruments against the concepts important to people living with MG.

Step 1 also included two waves of interviews conducted with individuals recruited from the Myaware MG patient association in the UK (www.myaware.org) based on self-reported MG diagnosis. In Wave 2, people with oMG or diagnosis of Lambert-Eaton myasthenic syndrome (LEMS) were excluded. Interviews were conducted over the telephone using a semi-structured interview guide. Ethical approval for the interviews in Step 1 was granted by the UK NHS Health Research Authority (https://www.hra.nhs.uk/).

Wave 1 of the interviews focused on concept elicitation to explore concepts important to patients; including their symptom experience and the impact of MG on their daily life. Analysis led to the development of a preliminary conceptual model of the patient experience in MG; generation of bespoke draft PRO items related to MG symptoms; and identification of the FATIGUE-PRO Physical Fatigue subscale as a candidate PRO instrument with content relevance in MG warranting further examination with participants [27].

Wave 2 of the interviews comprised both concept elicitation and cognitive debriefing, aiming to further build on the MG experience model while reviewing the draft items resulting from Wave 1 analysis as well as the FATIGUE-PRO Physical Fatigue items. Cognitive debriefing followed a ‘think-aloud’ process [28–30] to elicit spontaneous and probed feedback on the items` relevance, clarity, and ease of completion.

#### Step 2: MG0002 study exit interviews and PRO instrument completion

Face-to-face exit interviews were conducted locally by study personnel at the final study visit of the Phase 2 MG clinical study, MG0002 (ClinicalTrials.gov Identifier: NCT03052751), conducted across the US, Canada and Europe (Belgium, Czech Republic, Denmark, Germany and Spain) [26].

The objectives and design of the exit interviews were similar to those in Wave 2 of Step 1, comprising both a concept elicitation and cognitive debriefing section. MG0002 included people with moderate-to-severe gMG who were being considered for treatment with immunological therapy and had evidence of anti-AChR or anti-MuSK autoantibodies [26]. The draft MG Symptoms PRO was completed by MG0002 participants at 13 study visits during the treatment and observation periods. Ethical approval for the interviews in Step 2 was granted as part of the MG0002 study ethical approval/consent process.

### MMP analysis

We applied an MMP approach in Step 1 and Step 2, combining qualitative and quantitative analytic techniques, to generate evidence to inform item selection and refinement and to identify any anomalies in the item set [25].

#### Qualitative analysis

Interviews were transcribed verbatim and translated into English where applicable (22 of 36 interviews at Step 2 required translation). Thematic analysis was performed [31] with ATLAS.ti using a detailed, line-by-line, open and inductive coding approach [32–34]. Analytic techniques of conceptual model development were used to categorise the codes into higher order domains reflecting their underlying conceptual content [32, 33, 35]. Cognitive debriefing analysis involved multiple-level codes containing information on the corresponding item, response scale or instruction and the corresponding issue identified, findings of which were reviewed descriptively for each scale and item.

#### Item generation

Following both Step 1, Wave 1 and Step 2 analysis, new PRO items were generated based on the concept elicitation findings. Item generation followed item construction principles [24, 36–39], aiming to include an adequate range of items to cover the conceptual breadth within each of the target concepts of interest. Lay language and as many of the participants’ own words as possible were used, while aiming for brevity and minimal semantic overlap.

#### Quantitative analysis

The measurement properties of the PRO item sets were examined using both modern psychometrics (RMT) and traditional psychometrics (CTT). RMT analysis was used to first evaluate the measurement properties and make decisions on item selection and refinement, whereas CTT analysis was used to produce supportive evidence on the final PRO item sets.

RMT analysis was used to examine the measurement properties of each of the proposed draft item sets [40–42]. Specifically, it examined whether item response data achieved the requirements specified by the Rasch model in relation to (1) scale-to-sample targeting, (2) item response thresholds, (3) item fit, (4) item dependency and (5) person separation index (PSI). The principles of RMT analysis have been extensively described elsewhere [43]. We applied RMT analysis in two steps of the study design. First, a macro-level RMT analysis was performed on the data collected in the Wave 2 interviews of Step 1 to gain early insight on the item set. Second, a full RMT analysis was performed on the stacked data from all thirteen time-points of MG0002 in Step 2 using RUMM2030 software (RUMM Laboratory; Perth, Australia). In Step 2, RMT analysis was conducted in two rounds: First on the draft version of the scales; and, second on the available data of the revised version of the scales following the MMP results interpretations (Fig. 4). Stacked data were used to maximise the sample size of these analyses, which were repeated for comparative purposes on the first time-point of MG0002.

CTT psychometric analyses were further conducted on the MG0002 study data (Step 2) to generate complementary evidence for each scale of the PRO instrument that resulted from the RMT analysis. Investigated psychometric properties included reliability, both internal consistency (Cronbach’s alpha coefficient) and test–retest reliability (intraclass correlation coefficients were calculated in various sample and at various time-points), and construct validity (association of the reviewed scales with other available clinical outcome measures: QMG, MG Composite, MG-ADL). CTT analyses were performed using SAS v9.4 (SAS Institute, Thousand Oaks, NC, USA).

#### Integration of qualitative and quantitative analyses

All decisions regarding item modification or selection were informed by both qualitative and quantitative results, according to a pre-defined frame of reference devised to guide the decisions according to the following criteria [27]:

- Comprehensiveness: Informed by the breadth of coverage by the item set of both the qualitative conceptual model and of the quantitative measurement continuum from the Rasch model;
- Targeting and item quality: Informed by the endorsement of the items by participants in the qualitative feedback and match between the distribution of items and persons and appropriate item fit in the RMT analysis;
- Conceptual uniqueness: Informed by the lack of overlap between items reported by participants in the qualitative feedback, and the spread of the items on the continuum and absence of local dependency in the RMT analysis; and
- Appropriateness of response scale: Informed by any issues raised with the ease of selecting a response option by participants in the qualitative feedback, and the ordering of the item respond thresholds in a successive manner in the RMT analysis.

## Results

### Sample characteristics

A total of 60 participants were recruited for the interviews conducted in Step 1 (30 participants for each wave). A further 43 participants were included from the MG0002 clinical study in Step 2 (Table 1), 36 of whom participated in the exit interviews.

**Table 1:** Participant demographics and disease characteristics

	STEP 1: Wave 1 N (%)	STEP 1: Wave 2 N (%)	STEP 2 N (%)
*Gender*			
Female	15 (50)	20 (67)	27 (63)
Male	15 (50)	10 (33)	16 (37)
*Age, years*			
Mean	64.2	66.9	51.9
SD	13.7	13.4	15.1
Range	26–85	24–84	25–81
*Region*			
UK	30 (100)	30 (100)	–
Canada	–	–	11 (26)
Europe	–	–	23 (53)
USA	–	–	9 (21)
*Ethnicity*			
Caucasian	29 (97)	29 (97)	39 (91)

### Conceptual model of the patient experience in MG

**Fig. 2:**
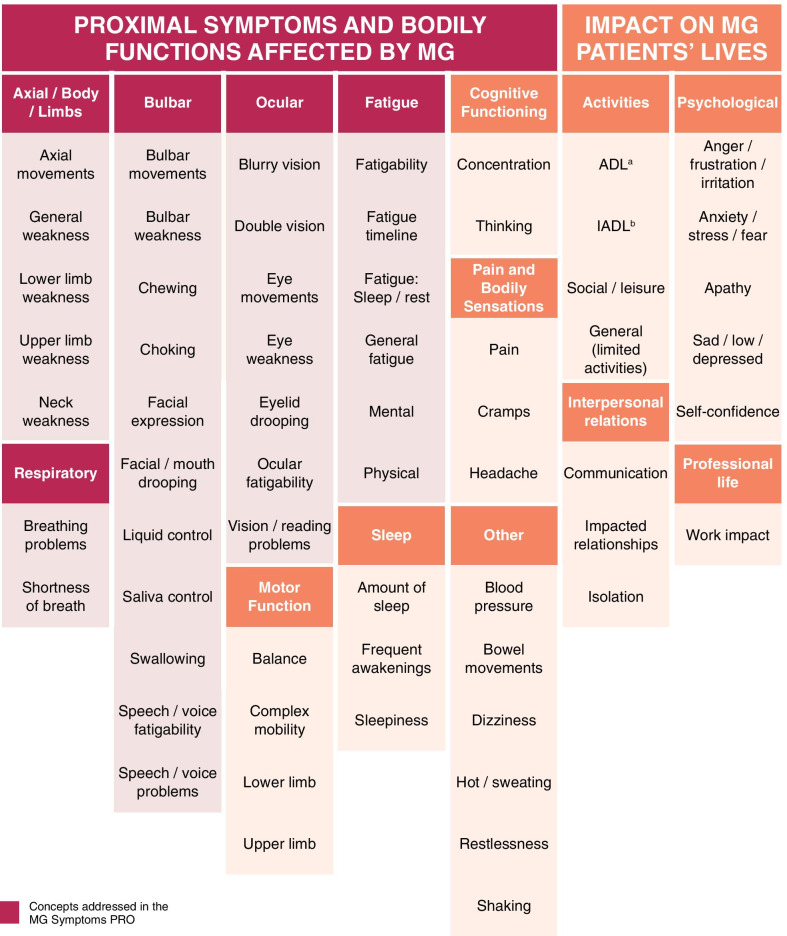
Conceptual model of the patient experience in MG. ^a^Activities of Daily Living (ADL) relates to routine activities including eating, bathing, dressing, toileting, transferring, and continence; ^b^Instrumental activities of daily living (IADL) are activities related to independent living such as preparing meals, shopping for groceries or personal items, performing light or heavy housework, doing laundry, and using a telephone

Concept elicitation analyses across both steps of the study resulted in a consolidated model of MG patient experience (Fig. 2). The model summarises the experience of living with MG in two overarching domains: Proximal symptoms and bodily functions affected by MG (i.e., disease-defining concepts, e.g., core signs and symptoms); and more distal impacts of MG on patients` lives (e.g., social functioning). The model was updated and refined at every step of research whilst the distinction of the proximal and distal concepts was informed by consultation with MG clinical experts.

Proximal symptoms were grouped into conceptual sub-domains in line with recognised MG symptomatology muscle groups, including ocular, bulbar and respiratory, as well as limbs axial and the entire body. Within each conceptual sub-domain examples of how muscle weakness manifests itself or affects bodily functions were included. For example, the bulbar sub-domain comprises a wide range of concepts, including general bulbar movements, facial drooping, saliva, liquid control, speech, and voice problems as well as chewing and choking. Concepts may also reflect different manifestations of each symptom and/or different severity levels of the symptom experience. The fatigue sub-domain appears to be relevant and proximal to the MG experience, particularly in relation to its physical manifestation. This is distinct from fatigability, which is a prominent proximal concept that can be relevant across symptoms and muscle groups for MG participants and is therefore present in multiple sub-domains in the model.

In addition, issues with motor and cognitive functioning were suggested by participants as relevant to their experience, as well as issues related to physical pain sensations and sleep, but these were deemed as less proximal upon consultation with the clinical experts. Lastly, a wide range of impact sub-domains were further identified with participants describing the impact of MG on their daily lives, from their basic daily activities to their instrumental, social and leisure activities, professional life, interpersonal relations, and feelings (psychological impact).

### Step 1: Cardinal concepts and item generation for the new MG PRO

#### Wave 1

**Fig. 3:**
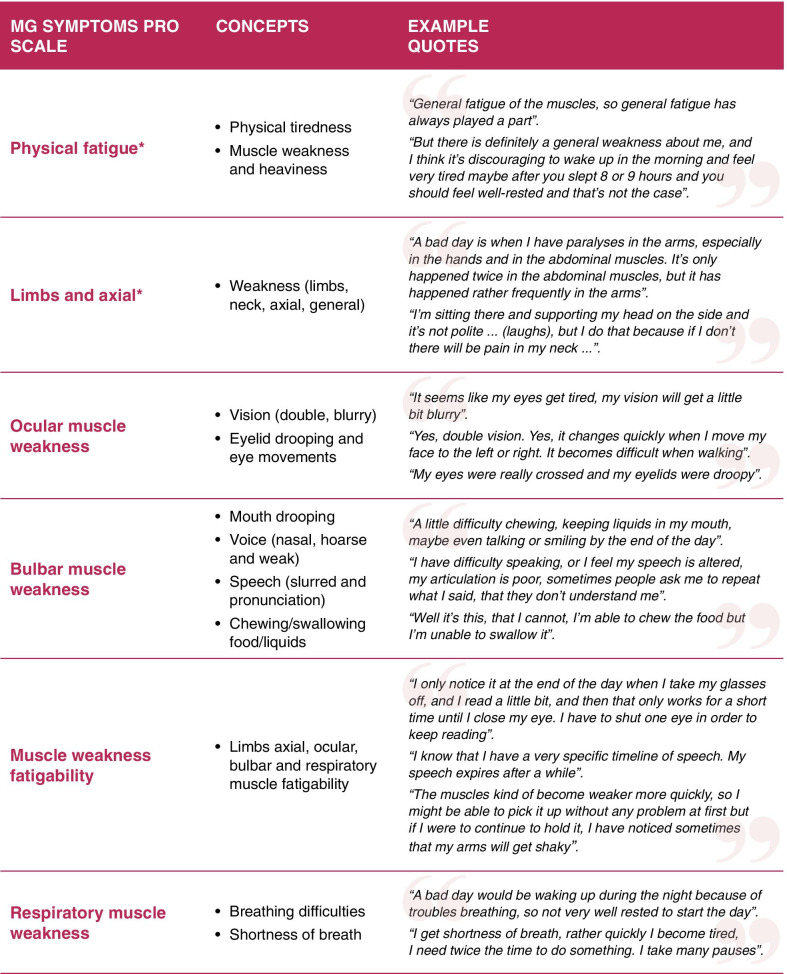
MG Symptoms PRO domains and underlying concepts with example quotes from cognitive debriefing. *Limbs and Axial concepts merged with Physical Fatigue scale in the final MG Symptoms PRO scoring structure

Review of the conceptual model (Fig. 2) led to the identification of the cardinal concepts of the proximal MG experience related to weakness and functional issues of the limb and axial, ocular, bulbar and respiratory muscles, and muscle weakness fatigability related to them, as well as physical fatigue (Fig. 3). The content of existing PRO instruments, including the MG-ADL [19], MG-QoL-15 [21], MG-DIS [14] and MGFS [20] identified and reviewed in the literature review (data not shown) was compared against the cardinal concepts in a qualitative mapping exercise. This exercise revealed gaps in the coverage of the reviewed PRO instruments, which either focused on more distal concepts or did not capture the proximal symptom concepts comprehensively. On this basis, 21 muscle weakness items across four muscle groups (i.e., ocular, bulbar, limbs and axial, and respiratory), and nine muscle weakness fatigability items were generated. Review of the physical fatigue concepts indicated that FATIGUE-PRO [27], a PRO instrument originally developed for systemic lupus erythematosus, was conceptually relevant to the aspects of the physical fatigue experience described in the context of MG. The physical fatigue domain scale (16 items) of the FATIGUE-PRO was therefore selected for further examination in MG alongside the newly generated items.

#### Wave 2

**Fig. 4:**
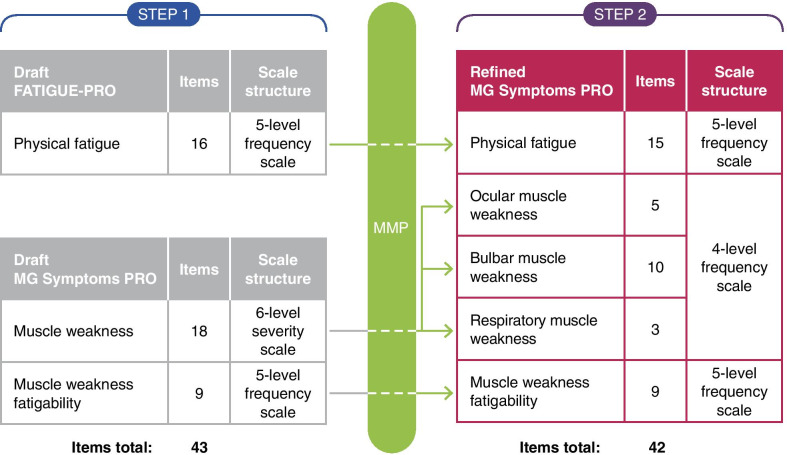
Item refinement of the MG Symptoms PRO. *MG* myasthenia gravis, *MMP* mixed methods psychometrics, *PRO* patient-reported outcome

MMP analysis indicated that the newly generated muscle weakness and muscle weakness fatigability items were clear, relevant, and easy to complete by people with MG. Some of the items, however, appeared to measure closely-related concepts (i.e., conceptual overlap), such as ‘pronouncing words’ and ‘slurred speech’, ‘nasal’ and ‘hoarse’ voice, and ‘swallowing’ and ‘controlling liquids in mouth’. Response scale issues were also identified, where participants were unable to distinguish accurately between the six different response options, particularly between ‘very mild’ and ‘mild’ options. The macro-level RMT analysis identified further issues related to potential conceptual overlap or uniqueness, item quality and appropriateness of response scale. Considering the small-scale basis of this analysis, the six-level response scale was retained with the plan to make a final decision on this issue at Step 2 of the work. Three items related to ‘aching’ were nonetheless deleted in response to clinical expert feedback in relation to their lack of specificity with MG pathology. A further item was rephrased to improve its clarity and interpretability resulting in the draft MG Symptoms PRO comprising two domain scales comprising 27 items: ‘Muscle weakness’ across ocular, bulbar, limbs and axial, and respiratory muscle groups (6-level severity scale; 18 items) and ‘muscle weakness fatigability’ (5-level frequency scale; 9 items) (Fig. 4).

The FATIGUE-PRO Physical Fatigue scale items were well-received with minimal interpretation or relevance issues and the macro-level RMT analysis demonstrated excellent targeting of this scale to the MG participants; therefore all 16 items (5-level frequency scale) were included in Step 2 analyses.

### Step 2: Psychometric evaluation of draft scales and PRO instrument refinement based on MMP evidence

#### Comprehensiveness

Participants did not specifically suggest that any symptom concepts were missing during the debriefing section; however, a qualitative comparison of item content against the refined conceptual model (Fig. 2) indicated minor gaps within the ocular and respiratory muscle weakness, which could be addressed with further item generation. Quantitative analyses showed good coverage of the targeted concepts in the participant sample (Additional file 1). Based on these findings, and in consultation with clinical experts, four additional items were generated: two related to ocular muscle weakness and two related to respiratory muscle weakness (Fig. 4)﻿.

#### Targeting and quality of items

Qualitative findings were supportive of the relevance, clarity, and ease of completion of items. Participants endorsed the relevance of the draft MG Symptoms PRO item content: On an individual item basis, items were found to be relevant to 86–100% of the sample. A few items related to bulbar symptoms proved not to be relevant for up to 14% of participants. This is in line with measurement development principles, aiming to generate item content reflective of different severity levels of the underlying construct of measurement, as well as clinical expectation of the increased relevance of bulbar symptoms in cases of higher MG disease severity. Findings for the FATIGUE-PRO physical fatigue scale were equally supportive, with items relevant to 86–100% of participants.

Quantitative RMT results (Table 2) indicated that all draft scales had good targeting, demonstrating the relevance of the item content in this population. No issues were identified with either the recall period or the instructions of these scales and there were few issues of clarity and interpretation; the draft MG Symptoms PRO items were found to be conceptually clear and unambiguous to 94–100% of the participants. A few items also showed some misfit to the Rasch model (Table 2).

**Table 2:** Summary of quantitative RMT measurement properties and findings

	Item #	Tageting^a^	PSI^b^	Disordered thresholds^c^	Item misfit^d^	Dependency^e^
*STEP 2: Draft scales*						
FATIGUE-PRO: Physical fatigue	16	91%	0.94	0%	13%	7
Muscle weakness	18	92%	0.90	33%	11%	15
Muscle weakness fatigability	9	89%	0.86	22%	33%	3
*STEP 2: Refined scales*						
Physical fatigue	15	89%	0.95	0%	13%	6
Ocular muscle weakness	3 + 2^f^	82%	0.57	0%	33%	0
Bulbar muscle weakness	9	56%	0.81	10%	20%	2
Muscle weakness fatigability			Same as draft			
Respiratory muscle weakness	N/A	N/A	N/A	N/A	N/A	N/A

*N/A* not applicable, Rasch measurement theory

^a^Percentage of sample measurements covered by the scale: The higher percentage, the better targeting

^b^Person Separation Index (PSI) ranges from 0 to 1: Higher scores reflect higher reliability

^c^Percentage of items displaying disordered response thresholds: Higher values suggest problems with the response scale

^d^Percentage of items displaying statistical misfit on the basis of Chi-Square values summarising the difference between observed and expected scores, where Chi Square significance is estimated with a Bonferroni adjustment at *p* < 0.01

^e^Number of item pairs displaying item dependency in the form of residual correlations > 0.30

^f^Two new items generated within Step 2; quantitative data are not yet available for these items

#### Conceptual uniqueness of items and scales

Qualitatively, some conceptual overlap was suggested, particularly in the draft MG symptoms PRO bulbar items, where 3–28% of participants indicated conceptual overlap of four different items, and within the FATIGUE-PRO physical fatigue items, where 3–17% of participants indicated overlap with six items. Some item dependency issues were also identified in the quantitative analysis, suggesting potential overlap/redundancies in the content of the items, as well as potentially more than one concept underpinning these scales. Based on these findings, it was decided that the muscle weakness scale would be revised to move away from a single total score of muscle weakness and instead use standalone domain scales reflecting each of the different muscle groups. This would better reflect the heterogeneity of MG pathology, which was indicated by item fit issues, as well as the qualitative and clinical expert information. In addition, considering the relative relevance and overlap between the FATIGUE-PRO scales and the newly generated draft MG Symptoms PRO scales, it was decided to merge the FATIGUE-PRO physical fatigue scale with the limbs and axial items of the muscle weakness scales.

#### Appropriateness of response scale

Minimal issues were raised with the response scale during the interviews: Only one participant raised issues with selecting a response option for five of the draft MG Symptom PRO items. No response scale issues were identified for the FATIGUE-PRO physical fatigue scale. RMT analysis also uncovered some issues with the ordering of the item response thresholds, particularly with the muscle weakness 6-level severity scale, where more than a third of the items displayed disordering, suggesting participants could not distinguish between six unique levels of severity for these items in the draft MG Symptoms PRO. Based on these findings, and in consultation with clinical experts, the muscle weakness items` severity response scale was reduced to four levels (Fig. 4).

### Step 2: Psychometric evaluation of the refined version of the PRO instrument

**Fig. 5:**
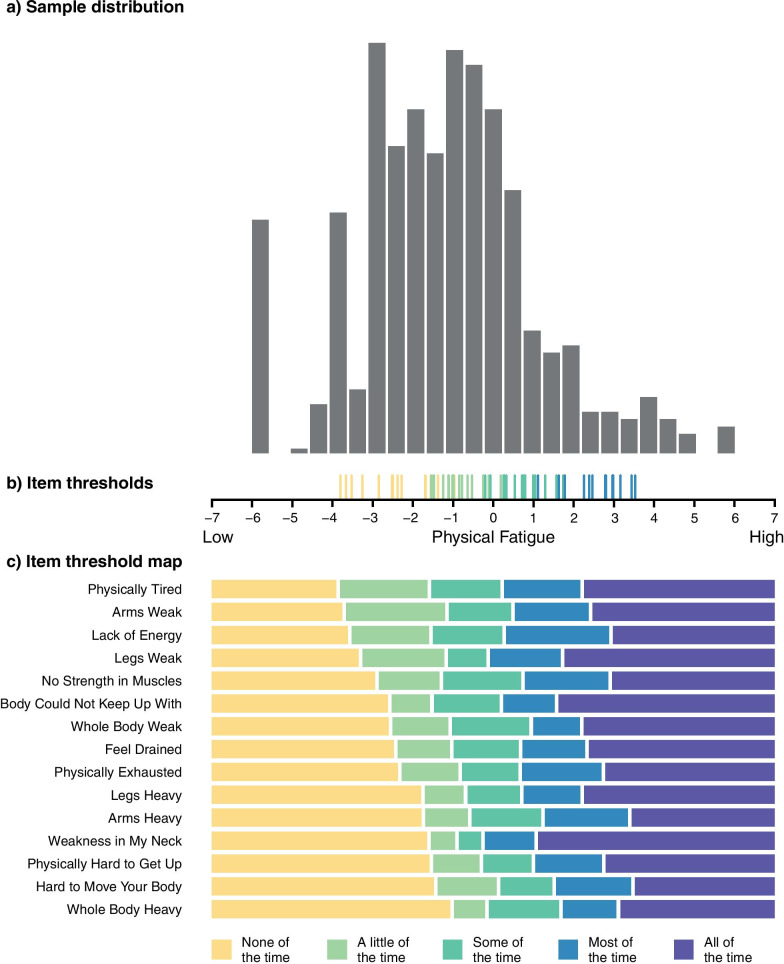
Exemplar sample-to-scale targeting plot—RMT analysis results for the Physical Fatigue scale. This figure depicts the person-item threshold distributions for the MG Symptoms PRO Physical Fatigue scale score, with persons (sample) distribution on top and scale item threshold distribution plotted on the same linear measurement continuum of physical fatigue. The sample distribution **a** represents the total score estimates for the physical fatigue scale plotted on a continuum of physical fatigue severity ranging from left (low severity) to right (high severity). The five-category response scale leads to four thresholds for each item. Therefore, the item threshold distribution **b** represents each of the four thresholds estimates for each item, plotted on the lower end of the same measurement continuum of physical fatigue. A threshold reflects the location on the measurement continuum where two adjacent response categories are equally likely to be endorsed. Targeting is assessed by examining the relative range and coverage of the sample distribution by the available item thresholds. The lower part of the figure **c** depicts the 15 items of the physical fatigue scale in the y-axis in order of increasing difficulty from top to bottom. The x-axis represents the most probable of the five response categories in the different coloured blocks across the range of the physical fatigue continuum. RMT expects the ordering of the response categories to reflect the intended severity i.e., from none of the time to all the time

The outcome of the MMP steps described above was a refined version of the MG Symptoms PRO instrument (Fig. 4). A final round of psychometric evaluation was performed for this refined PRO instrument (Table 2) demonstrating supportive overall results for all scales (Fig. 5; Additional file 1).

Targeting analyses demonstrated that the scales covered a good range of the participant sample locations (Additional file 1). Figure 5 shows the RMT analysis results for the Physical Fatigue scale, as an example. Only the bulbar muscle weakness scale had a relatively narrow coverage and larger floor effects, suggesting that items were not as relevant to participants with lower disease severity, which is in line with clinical expectations of bulbar symptoms (Additional file 1).

The person separation indices were high for all scales except the ocular muscle weakness scale (Table 2) which could be linked to the relatively small item number (n = 3) in the available data, but also to the specific composition of the sample, which excluded patients with ocular symptoms alone. The refined four-level severity scale of the muscle weakness items largely resolved the disordering of the original response scale with disordering being limited to one item. However, some item misfit and dependency issues persisted, particularly for the bulbar muscle weakness and physical fatigue scales (Fig. 5; Additional file 1).

Relative to the 18-item muscle weakness draft scale, the refined bulbar muscle weakness scale demonstrated sub-optimal targeting, and the ocular muscle weakness scale demonstrated reduced reliability. However, these findings were in line with clinical and measurement expectations. As bulbar symptoms are linked with higher levels of disease severity, the sub-optimal targeting of the scale for people with lower disease severity levels aligns with clinical expectation. The limited number of items of the ocular scale could further contribute to the reduced reliability of the scale. However, the generation of two additional ocular items could likely lead to improvement in the scale`s reliability, as shown by the conceptual clarity gained by refined muscle-group-specific scales in the context of a heterogenous condition, as opposed to the draft versions where all of these were part of a single muscle weakness score.

CTT results were also supportive of all revised scales, with good to excellent reliability demonstrated for most scales. Internal consistency (Cronbach’s alpha coefficients ranged from 0.70 to 0.95) with the scale comprising the fewer items; ocular muscle weakness showing the lowest reliability coefficient. Test–retest reliability coefficients were also supportive, particularly between study visits 13 and 15 (range 0.78 to 0.97) (Additional file 1). The correlations of the MG Symptoms PRO scale scores with clinician-reported measures (i.e., QMG, MGC) and the MG-ADL were at best moderate, which was expected given the difference in the targeted concept (Additional file 1).

## Discussion

We have developed a new MG-specific PCOM, the MG Symptoms PRO, using mixed methods evidence generated across 103 people living with MG, combined with sustained interactions with clinical experts and regulatory agencies. This new PRO instrument comprises 42 items across five scales: ocular-, bulbar-, and respiratory muscle weakness, physical fatigue, and muscle weakness fatigability—all rated on a recall period of 7 days (Fig. 4). The scales were purposefully designed as standalone to enhance score interpretation, and to allow for modular use (each scale can be used independently, depending on the specific concept of interest to be measured), given the heterogeneity of MG.

Compared with currently available PRO instruments, such as the MG-ADL [19], MG-DIS [14], MGFS [20], MG-QoL-15 [21] and MG Impairment Index (MGII) [44], the MG Symptoms PRO benefits from wider conceptual coverage and more patient-centred test design. Specifically, the MG Symptoms PRO contains more granular content and a detailed assessment of muscle weakness across different muscle groups, elaborate assessment of muscle weakness fatigability, as well as specific assessment of physical fatigue not currently included in other PRO instruments [22].

Unlike currently available PRO instruments, the development of the MG Symptoms PRO incorporated patient input at every stage, in line with regulatory and expert guidance for PCOMs [23, 24]. The MG Symptoms PRO further benefits from the application of the MMP approach and incorporation of complementary quantitative evidence, early in the item development process. This helped inform decisions on: Item and response scale refinement; scoring structure; and further demonstrated the strengths of the instruments` measurement properties early in the development process.

In a rare disease context, MMP proved to be a nimble and powerful approach to define and then refine a clinically meaningful set of items to assess MG severity. The extensive qualitative research has helped to provide a better understanding of MG, with a clear conceptualisation of the patient experience. The extensive qualitative patient input has ensured that the MG Symptoms PRO contains items covering all concepts relevant to the patient experience of MG, and worded in an appropriate way, whilst removing items linked to less relevant concepts. Moreover, the quantitative RMT analyses demonstrated the measurement robustness of the MG Symptoms PRO.

This study has three main limitations. First, although screening/inclusion criteria were applied to participants recruited for Step 1 of the work, no diagnostic confirmation of MG status was provided for participants as Myaware UK is a small patient advocate group. We aimed to correct for this by expanding our concept elicitation research in a clinically-defined sample [26]. Second, the item ‘neck weakness’, which is scored on a severity response scale, was included in the draft 18-item ‘muscle weakness scale’ in Step 1 but was moved to the ‘physical fatigue’ scale in Step 2, where other items are assessed by a frequency response scale. Third, the development and generation of early psychometric evidence on the MG Symptom PRO was performed in a relatively older, Caucasian sample, where mean ages in Step 1 were 64.2 years (range 26–85) and 66.9 years (range 24–84) for waves 1 and 2 respectively. The MG Symptoms PRO would benefit from further evaluation in participants of a wider age range, different socioeconomic status, and from different ethnicities or cultures.

Whilst the MG Symptoms PRO has an improved conceptual coverage as well as test design compared to other PRO instruments used in MG (MG-ADL, MG-DIS, MGFA, MG-QoL-15), it still requires further validation. Our currently available results indicated some outstanding conceptual overlap between items of the bulbar muscle weakness scale and other gaps for the measurement of ocular symptoms and respiratory symptoms. Four additional items have been developed to bridge those gaps and more data are needed to document their measurement performance, as well as explore possible refinement of the scales by excluding conceptually redundant items. For this purpose, the MG Symptoms PRO is being used in clinical studies to provide more data on the instrument. The next stage of this research will involve gathering additional qualitative and quantitative evidence on items generated following Step 2 and further exploration of some of the less optimal findings, as well as exploration of clinical meaningful change thresholds for this PRO instrument.

## Conclusion

In comparison to currently available PRO instruments used in MG, the MG Symptoms PRO contains more granular content and a detailed assessment of muscle weakness and muscle weakness fatigability symptoms, presented in a simple patient-centred way. This instrument also includes a detailed assessment of physical fatigue, an aspect of generalised fatigue not included in other PRO instruments. The MMP approach has allowed enhanced interpretation of not only item suitability, but also scale appropriateness. Most importantly, this instrument was developed with input from people with MG throughout the whole process leading to an instrument that is truly patient-centric, from the development of a conceptual model of MG through to the design of the actual instrument, including item terminology, item appropriateness and responses levels. Considering our qualitative and quantitative findings, the MG Symptoms PRO instrument shows promise as a measure of the symptoms experienced by people living with MG. It has great potential for both demonstration of treatment benefits in a clinical trial context and monitoring of symptom severity in a clinical practice setting, benefitting from a modular scale structure which enhances assessment and interpretability of outcomes in a heterogenous condition such as MG. Finally, the rigorous MMP approach followed in the development of the MG-Symptoms PRO offers a strong methodological framework for the development of future fit-for-purpose PRO instruments in the context of rare disease.

## Supplementary Information

**Additional file 1.** Quantitative RMT and CTT results. Threshold distributions and CTT analysis results for original and revised scales.

## Data Availability

Data sharing from non-clinical studies is outside of UCB’s data sharing policy, therefore the datasets used and/or analysed during development of the MG Symptoms PRO are available from the corresponding author on reasonable request. However, data from the MG0002 clinical trial may be requested by qualified researchers 6 months after product approval in the US and/or Europe, or global development is discontinued, and 18 months after trial completion. Investigators may request access to anonymised individual patient-level data and redacted trial documents which may include: Analysis-ready datasets, study protocol, annotated case report form, statistical analysis plan, dataset specifications, and clinical study report. Prior to use of the data, proposals need to be approved by an independent review panel at www.Vivli.org and a signed data sharing agreement will need to be executed. All documents are available in English only, for a prespecified time, typically 12 months, on a password protected portal.

## Declarations

### MG Symptoms PRO Copyright and Use

The copyright to the MG Symptoms PRO is owned by UCB Biopharma SRL, Belgium.

## References

[1] Morel T, Cano SJ (2017). Measuring what matters to rare disease patients—reflections on the work by the IRDiRC taskforce on patient-centered outcome measures. Orphanet J Rare Dis.

[2] Kempf L, Goldsmith JC, Temple R (2018). Challenges of developing and conducting clinical trials in rare disorders. Am J Med Genet A.

[3] Nestler-Parr S, Korchagina D, Toumi M, Pashos CL, Blanchette C, Molsen E (2018). Challenges in research and health technology assessment of rare disease technologies: report of the ISPOR rare disease special interest group. Value Health.

[4] IRDiRC. Orphan drug development guidebook. Building block I415: development and use of patient-centered outcome measures (PCOM). 2020 Available from: https://irdirc.org/building-block-forms-development-practices/.

[5] Binks S, Vincent A, Palace J (2016). Myasthenia gravis: a clinical-immunological update. J Neurol.

[6] NIH Genetic and Rare Diseases Information Center. Myasthenia gravis 2020. Updated 2016. Available from: https://rarediseases.info.nih.gov/diseases/7122/myasthenia-gravis.

[7] European Medicines Agency. EU/3/20/2272 2020. Available from: https://www.ema.europa.eu/en/medicines/human/orphan-designations/eu3202272.

[8] Gilhus NE, Tzartos S, Evoli A, Palace J, Burns TM, Verschuuren J (2019). Myasthenia gravis. Nat Rev Dis Prim.

[9] Nair AG, Patil-Chhablani P, Venkatramani DV, Gandhi RA (2014). Ocular myasthenia gravis: a review. Indian J Ophthalmol.

[10] Wang L, Zhang Y, He M (2017). Clinical predictors for the prognosis of myasthenia gravis. BMC Neurol.

[11] Conti-Fine BM, Milani M, Kaminski HJ (2006). Myasthenia gravis: past, present, and future. J Clin Investig.

[12] Thomsen JLS, Andersen H (2020). outcome measures in clinical trials of patients with myasthenia gravis. Front Neurol.

[13] Chen YT, Shih FJ, Hayter M, Hou CC, Yeh JH (2013). Experiences of living with myasthenia gravis: a qualitative study with Taiwanese people. J Neurosci Nurs.

[14] Raggi A, Schiavolin S, Leonardi M, Antozzi C, Baggi F, Maggi L (2014). Development of the MG-DIS: an ICF-based disability assessment instrument for myasthenia gravis. Disabil Rehabil.

[15] Richards HS, Jenkinson E, Rumsey N, Harrad RA (2014). The psychosocial impact of ptosis as a symptom of myasthenia gravis: a qualitative study. Orbit.

[16] Khadilkar SV, Chaudhari CR, Patil TR, Desai ND, Jagiasi KA, Bhutada AG (2014). Once myasthenic, always myasthenic? Observations on the behavior and prognosis of myasthenia gravis in a cohort of 100 patients. Neurol India.

[17] Barnett C, Merkies IS, Katzberg H, Bril V (2015). Psychometric properties of the quantitative myasthenia gravis score and the myasthenia gravis composite scale. J Neuromuscul Dis.

[18] Silvestri NJ, Wolfe G, Tarsy D (2018). Myasthenia gravis: classification and outcome measures. Myasthenia gravis and related disorders.

[19] Wolfe GI, Herbelin L, Nations SP, Foster B, Bryan WW, Barohn RJ (1999). Myasthenia gravis activities of daily living profile. Neurology.

[20] Kittiwatanapaisan W, Gauthier DK, Williams AM, Oh SJ (2003). Fatigue in myasthenia gravis patients. J Neurosci Nurs.

[21] Burns TM, Conaway MR, Cutter GR, Sanders DB, Muscle Study G (2008). Less is more, or almost as much: a 15-item quality-of-life instrument for myasthenia gravis. Muscle Nerve.

[22] Barnett C, Bril V, Kapral M, Kulkarni A, Davis AM (2014). A conceptual framework for evaluating impairments in myasthenia gravis. PLoS ONE.

[23] Food and Drug Administration. Patient-focused drug development—collecting comprehensive and representative input 2020 updated June 2020. Available from: https://www.fda.gov/media/139088/download.

[24] Food and Drug Administration. Guidance for industry: patient-reported outcome measures: use in medical product development to support laveling claims 2009 [Available from: https://www.fda.gov/media/77832/download. 10.1186/1477-7525-4-79 PMC1629006 17034633

[25] Regnault A, Willgoss T, Barbic S, International Society for Quality of Life Research Mixed Methods Special Interest G (2018). Towards the use of mixed methods inquiry as best practice in health outcomes research. J Patient Rep Outcomes.

[26] Bril V, Benatar M, Andersen H, Vissing J, Brock M, Greve B (2021). Efficacy and safety of rozanolixizumab in moderate-to-severe generalised myasthenia gravis: a phase 2 RCT. Neurology.

[27] Cleanthous S, Regnault A, Haier B, Stach C, Cano S, Morel T (2019). A mixed methods psychometrics program for the development of a measure of fatigue in SLE. Qual Life Res.

[28] Blair J, Presser S. Survey procedures for conducting cognitive interview to prestest questionnaires: a review of theory and practice. In: JSM proceedings, survey reesearch methods section [Internet]. Alexandria, VA: American Statistical Association; [370–6]; 1993. Available from: http://www.asasrms.org/Proceedings/papers/1993_059.pdf.

[29] Streiner D, Norman GR, Cairney J (2015). Health measurement scales: a practical guide to their development and use.

[30] Kerr C, Nixon A, Wild D (2010). Assessing and demonstrating data saturation in qualitative inquiry supporting patient-reported outcomes research. Expert Rev Pharmacoecon Outcomes Res.

[31] Braun V, Clarke V (2006). Using thematic analysis in psychology. Qual Res Psychol.

[32] Bryman A, Burgess RG, Paulsen Chico N, Droes N, Evans K, Hatton D (1994). Analyzing qualitative data.

[33] Thomas DR (2006). A general inductive approach for analyzing qualitative evaluation data. Am J Eval.

[34] Bowling A (2014). Research methods in health: investigating health and health services.

[35] Klassen AF, Pusic AL, Scott A, Klok J, Cano SJ (2009). Satisfaction and quality of life in women who undergo breast surgery: a qualitative study. BMC Womens Health.

[36] Kline P (2015). A handbook of test construction: introduction to psychometric design.

[37] Feinstein AR (1977). Clinical biostatistics. XLI. Hard science, soft data, and the challenges of choosing clinical variables in research. Clin Pharmacol Ther.

[38] Fowler FJJ (1995). Applied social research methods series. Improving survey questions: design and evaluation.

[39] DeVellis RF (2017). Scale development: theory and applications.

[40] Andrich D (2011). Rating scales and Rasch measurement. Expert Rev Pharmacoecon Outcomes Res.

[41] Rasch G (1960). Probabilistic Models for some intelligence and attainment tests.

[42] Wright BD, Stone MH (1979). Best test design: Rasch measurement.

[43] Hobart J, Cano S (2009). Improving the evaluation of therapeutic interventions in multiple sclerosis: the role of new psychometric methods. Health Technol Assess.

[44] Barnett C, Bril V, Kapral M, Kulkarni A, Davis AM (2016). Development and validation of the myasthenia gravis impairment index. Neurology.

