# Supplementary material for: Development of the Myasthenia Gravis (MG) Symptoms PRO: a case study of a patient-centred outcome measure in rare disease
Source: Orphanet J Rare Dis. 2021 Oct 30;16:457. doi: 10.1186/s13023-021-02064-0 (PMC8556940; doi:10.1186/s13023-021-02064-0)
Supplement: Supplementary file 1 — Additional file 1. Quantitative RMT and CTT results. Threshold distributions and CTT analysis results for original and revised scales. [file 13023_2021_2064_MOESM1_ESM.pdf]

Quantitative RMT results

These figures depict the person-item threshold distributions for MG Symptoms PRO domains for bulbar symptoms, ocular symptoms, and muscle weakness fatigability. The upper histogram shows the person (sample) distribution for the scale total score estimates, plotted on a severity continuum ranging from left (low severity) to right (high severity). The lower histogram represents the scale item threshold estimate distribution, plotted on the same interval-level measurement continuum. A threshold reflects the location on the measurement continuum where two adjacent response categories are equally likely to be endorsed. RMT expects the ordering of the response categories to reflect the intended severity i.e., from none of the time to all the time

Quantitative RMT results of original scales

Bulbar muscle weakness: targeting

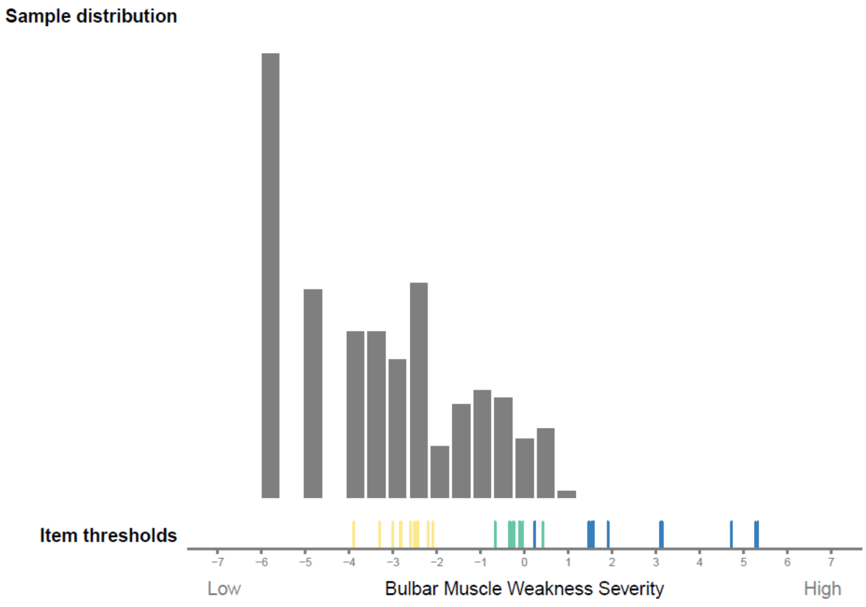

Ocular muscle weakness: targeting

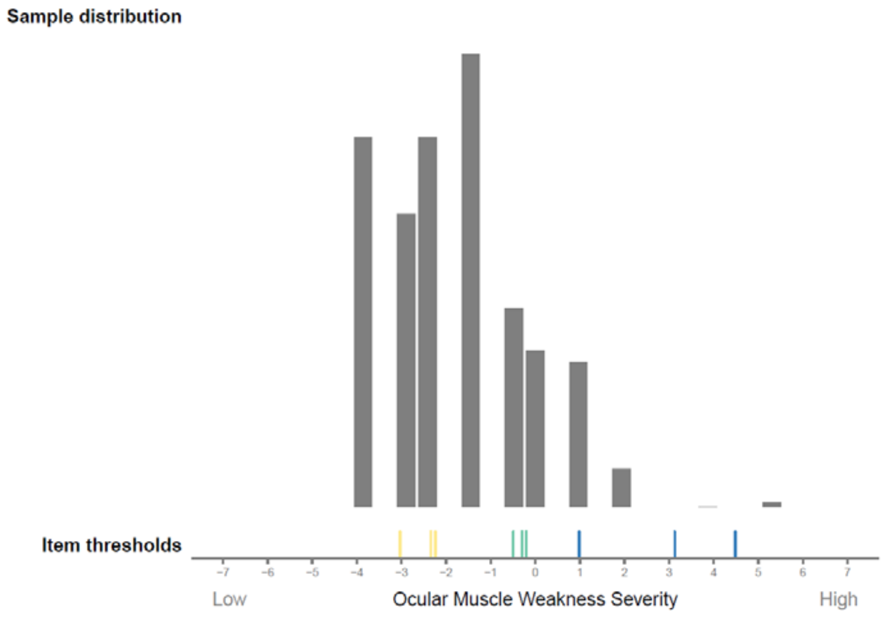

Muscle weakness fatiguability targeting

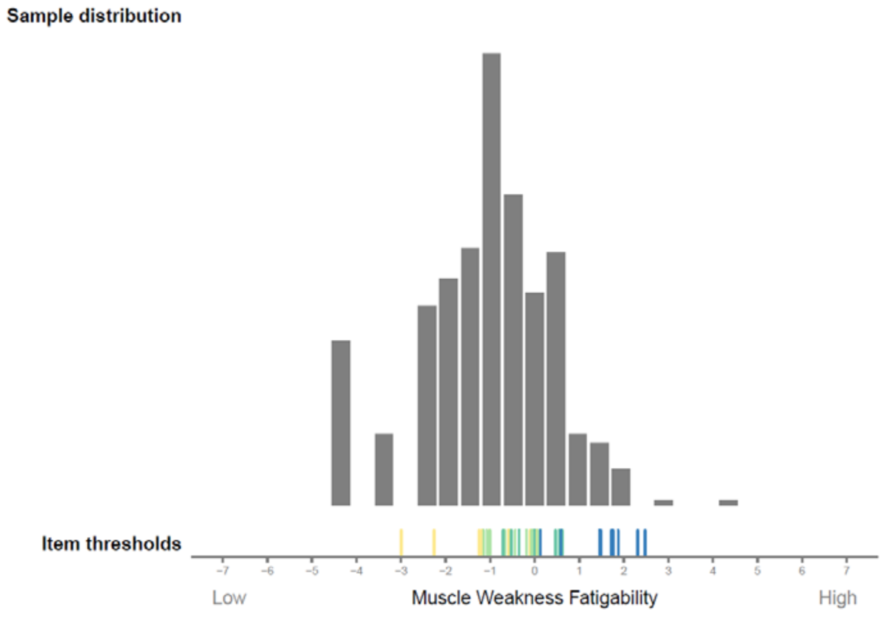

CTT analyses

CTT results were supportive of all revised scales. Good to excellent reliability was demonstrated for most scales. As expected, the scales with the fewest items (respiratory symptoms and ocular symptoms) showed the lowest reliability coefficients.

Reliability statistics for the MG Symptoms PRO

	Internal consistency reliability		Test-retest between Baseline and Visit 4		Test-retest between Visit 13 and Visit 15	
**Scale**	N	Cronbach's Alpha	N	ICC	N	ICC
**Ocular muscle weakness**	43	0.70	28	0.77	28	0.89
**Bulbar muscle weakness**	43	0.89	28	0.90	28	0.92
**Respiratory muscle weakness**	NA	NA	28	0.37	28	0.78
**Physical fatigue original form**	43	0.95	28	0.82	28	0.97
**Muscle weakness fatigability**	42	0.87	27	0.81	28	0.94

ICC, item characteristic curves; NA, not applicable.

Correlation of the MG Symptoms PRO scale domains with the MGC, QMG and MG-ADL scores at baseline.

MG Symptoms PRO scale (N)	Correlation coefficient*		
	MGC	QMG	MG-ADL
**Ocular muscle weakness (43)**	0.39	0.51	0.63
**Bulbar muscle weakness (43)**	0.50	0.46	0.62
**Respiratory muscle weakness (43)**	0.37	0.31	0.35
**Physical fatigue original form (43)**	0.20	0.34	0.40
**Muscle weakness fatigability (42)**	0.56	0.58	0.74

*** Spearman correlation coefficient.**
In white, low correlations (<0.30); in light blue, mild correlations (0.30–0.49); in dark blue, moderate correlations (0.50–0.79).
